# Macromolecular X-ray crystallography: soon to be a road less travelled?

**DOI:** 10.1107/S2059798320004660

**Published:** 2020-04-30

**Authors:** John H. Beale

**Affiliations:** a Swiss Light Source, Paul Scherrer Institut, 5232 Villigen, Switzerland

**Keywords:** macromolecular X-ray crystallography, protein crystallization, Protein Data Bank

## Abstract

The number of new X-ray crystallography-based submissions to the Protein Data Bank appears to be at the beginning of a decline, perhaps signalling an end to the era of the dominance of X-ray crystallography within structural biology. This letter, from the viewpoint of a young structural biologist, applies the Copernican method to the life expectancy of crystallography and asks whether the technique is still the mainstay of structural biology. A study of the rate of Protein Data Bank depositions allows a more nuanced analysis of the fortunes of macromolecular X-ray crystallography and shows that cryo-electron microscopy might now be outcompeting crystallography for new labour and talent, perhaps heralding a change in the landscape of the field.

## Abstract 2

From the perspective of a young(ish) structural biologist who currently specializes in macromolecular X-ray crystallography, are the best years of crystallography over? Some evidence and hopefully thought-provoking analysis on the subject is presented here.

As a young structural biologist specializing in macromolecular X-ray crystallography (MX), I have become increasingly reflective upon the mileage of the technique and, assuming that I want to, whether remaining in the field for my entire career will even be possible. Could my career perhaps outlast X-ray crystallography? These musings were given more teeth recently when I was introduced to the ‘Copernican method’, originally formulated by J. R. Gott (Gott, 1993 ▸). This method gives a framework for how to estimate the probable lifespan of something given no prior information other than how long something has already existed. The Copernican method assumes that the probability of observing anything during its lifespan can be normally distributed [Fig. 1 ▸(*a*)]. An unknowing observer witnessing a phenomenon at any particular point of its lifespan does so accidentally. Given this, and in the absence of any other pertinent information, inferences can be drawn about the probable duration of the phenomenon based purely upon the time that has elapsed between the inception of the phenomenon and the time of observation. Applying features of the normal distribution to this concept, at any moment when a phenomenon is observed there is a 68.27%, 95.45% and 99.73% chance that the observation occurred within one, two and three standard deviations (μ), respectively, from the mean time of observation within its lifespan (σ). The key to applying the method with any degree of certainty is to know the start date of the phenomenon of interest. Gott originally applied his method to the question of how long the Berlin Wall would stand and to the lifespan of the human race (Gott, 1993 ▸; Ferris, 1999 ▸), subjects that have at least a reasonably clear physical beginning.

So, what happens when the Copernican method is applied to MX? When thinking about the beginning of MX, I am drawn to three debatable times in human history: 1839, the first known case of protein crystallization (Hünefeld, 1839 ▸); 1934, the first recording of protein diffraction (Bernal & Crowfoot, 1934 ▸); and 1960, the first high-resolution protein structure (Kendrew *et al.*, 1960 ▸). Table 1 ▸ contains the results of applying the Copernican method to these different beginnings and leads to relatively broad end ranges, perhaps somewhat pleasing for someone wanting to stay in the field for a few decades yet. Even given a start date of 1960 for MX, there is approximately a 70% chance that it will last another 11–202 years. Given the nonlinearity of the future trends (Tetlock & Gardner, 2015 ▸), forecasting the end of MX with any degree of certainty higher than this range is liable to make the predictor look foolish in years to come. On the other hand, the breadth of these estimates are rather unsatisfying and the results are rendered practically useless because of this. So, at the distinct risk of present and future ignominy, let me throw accuracy to the wind and brazenly attempt more precision. Gott’s principle is designed to be used in situations where nothing else can be known about the subject but, in the case of MX, we undoubtedly know rather a lot.

Instead of using the Copernican method, it is also possible to think about the lifespan of a subject in terms of a normally distributed population. For example, when considering the lifespan of the human race, instead of using Gott’s method of normally distributed potential observations of the human race, one could use the frequency of individual human existences. This has been demonstrated by Leslie (1992 ▸) to come to a far more sobering estimate of human existence than that of Gott’s estimate. As a means of analysing the lifespan of a particular technology or technique, such as MX, this method, however, carries with it some challenges. Not least, how is one to measure the frequency of the application of the technology or method, *i.e.* a ‘population’ to describe its existence? Also, assuming that an acceptable measure can be found, what are we defining precisely as the ‘technology of interest’? For example, in the case of computing, would you class computers in their entirety as a piece of technology, where their use is debatably still growing, or as individual components? For instance, particular types of chips, as standalone products in their application, have been shown to follow Gaussian lifespans (Bayus, 1998 ▸). Furthermore, this way of analysing the lifetime of a methodology, such as MX, is complicated by the fact that methods used within MX ebb and flow in popularity or relevance and may not follow a normal distribution themselves. For example, merging data from many hundreds of crystals was not regularly practised in MX after the widespread adoption of cryo-methods until the dawn of free-electron lasers (XFELs) in 2009 (Powell, 2019 ▸).

With these caveats in mind, let us instead explore the lifespan of MX and the other structural biology methods using Protein Data Bank (PDB; https://www.ebi.ac.uk/pdbe/) depositions as our measure of population. When I began my PhD research in 2010, MX was always presented to me as being in a period of exponential growth. However, approximately ten years on, the growth in annual PDB depositions from MX appears to be slowing and perhaps even beginning a prolonged decline.

Fig. 1 ▸(*b*) shows the number of PDB depositions from 1976 to 2019 for the three principal structural determination techniques: NMR, MX and electron microscopy (EM). Although MX is still the dominant technique in terms of PDB depositions, accounting for 89% of all PDB entries up to the end of 2019, the year-on-year growth observed since the 1980s (with the exception of 2015) has for the first time seen two consecutive years of decline. By comparison, the two other principal methods of structural biology potentially show a vision of Christmas past and future for MX. Depositions from nuclear magnetic resonance (NMR) spectroscopy have been in decline since 2007, but we are now seeing exponential year-on-year growth in structures determined by EM. Figs. 1 ▸(*c*) and 1 ▸(*d*) show Gaussian fits to the frequency of NMR and diffraction-based depositions, respectively.^1^ The NMR fit, in particular, shows that normal distributions do not provide a perfect model for these kind of analyses. The fit to the MX depositions is also precarious given the lack of data on the descending tail of the model. However, putting these objections to one side, these models based on PDB depositions have given us two precise estimates for the end of NMR-based and MX-based depositions of 2036 ± 2 and 2063 ± 1, respectively. Even given my scepticism of this exercise, it is still sobering to see these numbers and to contemplate their implications. In the case of MX, perhaps the 121 996 structures submitted by 2017 represent more than 50% of all of the depositions that will ever be submitted using MX, and 2019 marks the beginning of an inevitable decline, mirroring the decline observed in NMR depositions. From a personal perspective, an end to MX as we know it by 2063 might just allow for my retirement (assuming that by then the retirement age has not been increased to 80 years of age), but it is nail-bitingly close.

There are at least conceptual reasons for the observed apparent decline in MX and the increase in EM, and these do not necessarily mean that MX is but the corn to EM’s sickle. The ‘resolution revolution’ in EM (Kühlbrandt, 2014 ▸), the accessibility of microscope facilities, and numerous other advances in computing and software have helped to expand the EM user base and have resulted in a dramatic increase in output efficiency. The decline in MX PDB depositions may also reflect the end of the era of ‘low-hanging fruit’ in MX and, as projects become more challenging, more resources are required to generate the same level of output. This latter point can best be observed by the development of serial crystallo­graphy at XFELs (Johansson *et al.*, 2017 ▸) and now also electron diffraction (for recent reviews, see Clabbers & Abrahams, 2018 ▸; Nannenga & Gonen, 2019 ▸). These methods have further enabled the collection of diffraction data from submicrometre crystalline samples and, in the case of serial crystallography, have made room-temperature, time-resolved crystallography experiments a reality. All of the above might explain the trends observed in Fig. 1 ▸ without necessarily proving that a changing of the guard is in progress. However, an analysis of the pool of ‘new PDB authors’ suggests that not only is EM output growing, but also that EM-based research is a powerful attraction to new structural biologists.

The ability to attract new labour and talent is an interesting yardstick for the health of a discipline. As ideas and output grow, ever more people are likely to be drawn into their orbit. When the labour-force increase starts to wane, this perhaps signals that growth in output is also starting to taper off, and the different stages of this ‘boom-and-bust’ cycle can be seen in structural biology (Fig. 2 ▸). The field as a whole is still generally seeing year-on-year growth in the number of PDB entries; however, the yearly pool of new structural biologists, as measured by ‘new PDB authorships’, may have peaked [Fig. 2 ▸(*a*)], and has for the first time dropped below the number of new depositions. When this overall trend is broken down by method, we gain a better understanding of how structural biologists are behaving. Figs. 2 ▸(*b*), 2 ▸(*c*) and 2 ▸(*d*) show the data in Fig. 2 ▸(*a*) broken down by method: NMR, MX and EM, respectively. NMR is a technique that appears to have already peaked in popularity and is currently in a period of decline in terms of its PDB output. Interestingly, the peak in NMR-based PDB depositions in 2007 was preceded by a peak in new NMR authors, which occurred in 2003. As the number of new authors declined, a comparable decline in PDB output was also observed.

EM, in contrast, is at the beginning of its life cycle. Both in terms of new labour and new output, EM is growing exponentially as a technique, taking more structural biologists under its wing. MX appears to show a trend closer to that of NMR, rather than that of EM. The number of new crystallo­graphers peaked in 2012 and appears now to be declining, reinforcing the prediction from Fig. 1 ▸(*d*) that MX has seen its most productive days and is now in decline. It is also possible, if it is assumed that there is a finite pool of yearly new talent within structural biology, that the recent successes of EM are drawing researchers away from MX and that this has contributed to the decline in MX PDB output. There is also some evidence to suggest that the pool of new structural biologists is decreasing. An analysis of jobs posted on the Collaborative Computational Project Number 4 Bulletin Board (CCP4BB) suggests that since 2009 there has been a general reduction in the number of structural biology jobs [Fig. 3 ▸(*a*)]. This decline is likely to have been triggered by the global slowdown caused by the financial crisis in 2008. Fig. 3 ▸(*a*) also shows a breakdown of job postings that specifically mention MX or EM methods, and these data also seem to corroborate the results from the ‘new author analysis’ that EM is now providing more new opportunities for structural biolo­gists than MX.

However, it is worth remembering at this point the crudeness of using data such as CCP4BB job postings and PDB deposition frequencies as an overall guide to the health of a given structural technique. The CCP4BB was not designed to be a vehicle for job adverts (a subject the community has even debated; https://www.jiscmail.ac.uk/cgi-bin/webadmin?A2=ind0909&L=CCP4BBl&D=0&O=D&P=331308) and, although it is often used as such by structural biologists from both academia and industry, it may present an unrepresentative sample of opportunities within structural biology. With respect to PDB depositions, the data presented in both Figs. 1 ▸ and 2 ▸ paint a gloomy outlook for the present condition of NMR spectroscopy within structural biology. However, an equally blunt analysis of PubMed (https://www.ncbi.nlm.nih.gov/pubmed/) articles found using the search terms ‘structural biology’ and either ‘NMR’, ‘X-ray crystallography’ or ‘EM’ suggests that NMR is far from doomed [Fig. 3( ▸ *b*)], almost matching EM in its publication output. Since NMR can be applied to other research areas such as enzyme kinetics (Smith *et al.*, 2015 ▸) or protein folding (Zhuravleva & Korzhnev, 2017 ▸), publications on which tend not to yield a PDB deposition, this discrepancy is not surprising. PDB deposition and new PDB authorship frequencies will also be sensitive to external biases that might skew the data. For example, as the storage rings around the world upgrade to fourth-generation designs, there have been, and will continue to be, dark spells in synchrotron light. The user program at the European Synchrotron Radiation Facility (https://www.esrf.eu/about/upgrade) is due to restart in 2020 after a year of downtime. Has this downtime already been baked into the MX PDB deposition frequencies or are its effects still to be felt? Another consideration is nicely exemplified in Fig. 3 ▸(*c*), which plots the mean number of PDB authors and publications linked to PDB entries determined by EM methods. The gradual decrease in EM PDB authors observed since 2014 during a time of exponential publication growth suggests that more work is now being done by fewer people and that EM, as a field, has managed to make significant labour economies that have ultimately increased productivity. The same increase in productivity was not observable over this period for MX (data not shown) and this does seem surprising given all of the technological advances in MX data-collection and processing methods that have undoubtedly increased the potential output from a single person (Grimes *et al.*, 2018 ▸). However, it may simply be that crystallographers are more generous with their distribution of PDB authorships.

The individual PDB deposition yardstick used in this analysis may also be about to fundamentally change. Depositions in 2017 were significantly augmented by a dramatic number of structures submitted from ‘fragment-screening’ experiments. Fig. 3 ▸(*d*) plots the frequency of PDB depositions from structural genomics consortia (SGC) and the subgroup of these that are associated with the XChem facility (Collins *et al.*, 2018 ▸) based at I04-1, Diamond Light Source, UK. Fragment screening relies on the rapid measurement of hundreds of crystals with different ligand soaks and the submission of all of these individual structures to the PDB would entail a large amount of work, begging the question already posed by others: are multiple single structures the best way to make this information available to a wider scientific audience? Instead of submitting single protein structures, perhaps as the PDB evolves an ensemble of ligand-bound structures will become the norm. This kind of deposition would also have an impact on time-resolved studies, which are currently curated as a series of still frames. Both of these changes, if enacted, would dramatically increase the amount of work required for an individual ‘ensemble deposition’ and would fundamentally change the calculations used for these analyses.

Partly owing to all these considerations and partly owing to denial, I am personally inclined to be a bit more optimistic than the prediction of 2063 for the ‘final gasp’ of MX. Firstly, the ‘Copernican method’ is given its name because it is based upon the idea that we are not ‘special observers’ and that it would be extremely unlikely for us to observe any specific time in the lifespan of a particular phenomenon. Observing the exact middle of a lifespan is a special event; therefore, observing it, as predicted by the analysis presented here, would make me special and, as those who know me can soundly attest, I am not. Secondly, owing to the nature of these analyses, I have written about these structural biology techniques as if they are competing against one another, ever locked in a titanic struggle until judgement day and the trumpets sound. This is simply not the case, as all of these methods are highly complementary and their use is primarily driven by the scientific question at hand.

Some of the early successes in EM were in part based upon MX methods, since to gain the resolution required for bio­logical understanding, structures determined using MX had be fitted to the larger complex maps that were only determinable by EM (Lander *et al.*, 2012 ▸). It is as yet unclear whether this inter-dependence of methods will continue to be the case, or whether further developments in EM will jettison the need for any prior knowledge based on MX methods (or indeed NMR; Gauto *et al.*, 2019 ▸) and routine measurements of 2.0 Å resolution will be possible using EM (Cheng, 2018 ▸). However, it is likely, at least in the short term, that both past, present and future MX will continue to be able to provide essential information for future EM experiments (Higgins & Lea, 2017 ▸). It is also quite possible that EM could begin to provide essential information to improve MX. We are ultimately not talking about individual methods, but of structural biology and the structural biologists who practice it. Therefore, although I may currently specialize in MX methods, the reason I was drawn, and probably the reason many structural biologists were drawn, to this field was a deep interest in biological structure and function. For me, it is quite simply an exciting time to be a structural biologist.

To end, given the data presented here, a shift within structural biology is probably in progress and is probably being driven by the broadening utility of EM. Purely in terms of the people involved in the different methods, new structural biologists are becoming more likely to be placed in laboratories that are utilizing EM and they are therefore gaining experience of EM. This growth in people practising EM methods might overtake those in MX in number and utility, and make MX the less common path. However, to complete the thought that was suggested by the Robert Frost-inspired title of this letter, let me finish with a different quotation. When it comes to structural biology as a whole, I am more inclined to follow the advice of Yogi Berra: ‘When you come to a fork in the road, take it!’. That is to say, make the most of *all* the methods available which are appropriate to you and your scientific question, regardless of their age and popularity.

## Figures and Tables

**Figure 1:**
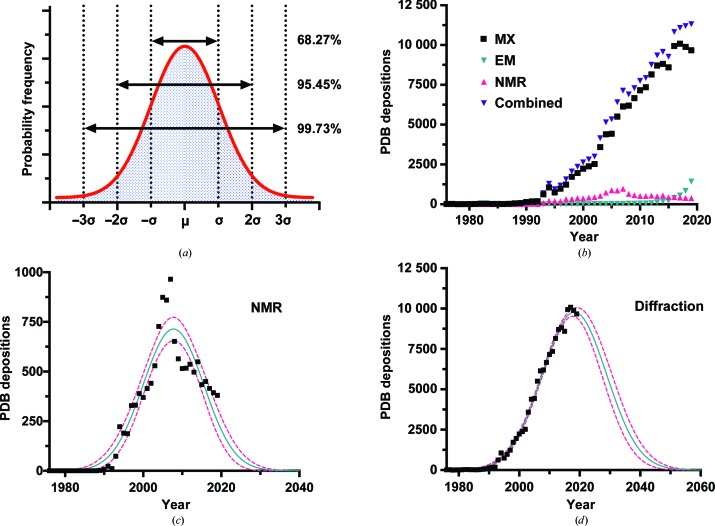
(*a*) A representation of the normal distribution, where the probability of an event happening is symmetrically dispersed about the mean (μ), with 68.27%, 95.45% and 99.73% of all events occurring within one, two and three standard deviations (σ), respectively, from the mean. (*b*) The frequency of PDB depositions, grouped by year, from MX, EM, NMR and all three techniques combined. (*c*, *d*) Gaussian fits (shown in green) of the depositions from NMR and MX, respectively. The 95% confidence limits are highlighted in pink.

**Figure 2:**
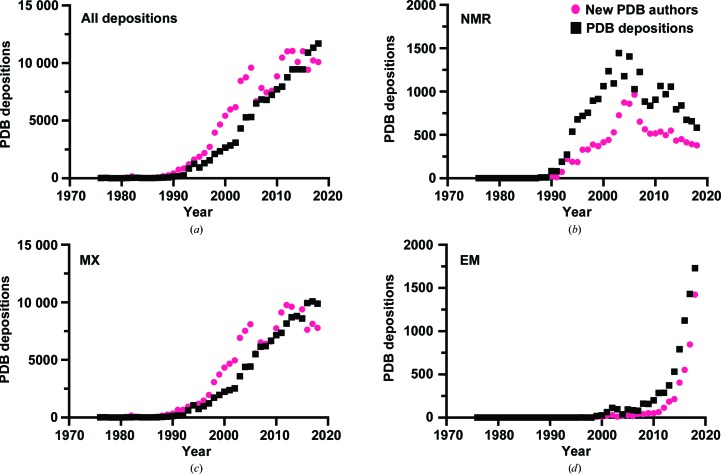
Frequency plots of ‘new authors’ from PDB depositions, grouped by year, for (*a*) all techniques, (*b*) only NMR, (*c*) only MX and (*d*) only EM. A ‘new author’ was defined as a PDB author name that does not appear in any previous deposition. The frequency of depositions per year is also plotted.

**Figure 3:**
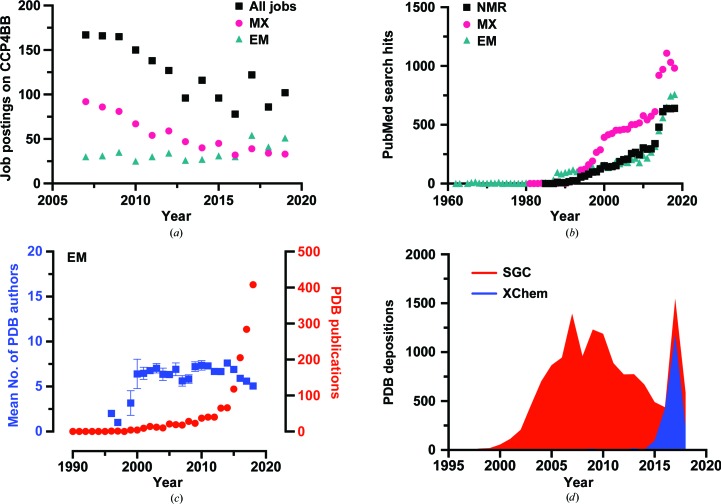
(*a*) The frequency of structural biology-linked job postings on the CCP4BB. A database of job postings was made using a broad keyword search in both the Subject and Content fields and then manually pruned. Job postings were sorted based on their inclusion of keywords associated with either MX or EM, such as ‘crystal’ and ‘electron’, respectively. (*b*) Frequencies, grouped by year, of research articles which were found by searching for the phrase ‘structural biology’ and either ‘NMR’, ‘X-ray crystallography’ or ‘EM’. (*c*) The mean number of PDB authors and publications connected to PDB entries from EM depositions. The systematic error of the mean (SEM) is depicted where practicable. (*d*) The frequency of PDB depositions from SGC-based organizations and a subgroup of those depositions linked to the XChem project at Diamond Light Source, UK.

**Table 1:** MX lifespan projections based upon the Copernican method The confidence intervals are based on a normal distribution [Fig. 1 ▸(*a*)]: 68.27%, 95.45% and 99.73% correspond to 1σ, 2σ and 3σ, respectively. The two-tailed estimates are on the limits of the confidence interval.

Confidence (%)	Hünefeld (1839 ▸)	Bernal & Crowfoot (1934 ▸)	Kendrew *et al.* (1960 ▸)
68.27	2054–2940	2035–2454	2030–2321
95.45	2023–2974	2021–5668	2020–4552
99.73	2019–135172	2019–64867	2019–45664

## Footnotes

1 A Gaussian fit of the EM depositions was not attempted, as my ability to suspend all my disbelief is not infinite.
